# What effects does peer support work have and how to implement it into forensic hospitals? A Review of Reviews

**DOI:** 10.1192/j.eurpsy.2024.1214

**Published:** 2024-08-27

**Authors:** P. Walde, B. A. Völlm

**Affiliations:** Forensic Psychiatry, Rostock University Medical Center, Rostock, Germany

## Abstract

**Introduction:**

Despite promising research there is still hesitation in implementing peer support work in some hospitals. Especially in forensic hospitals reservations are held against peer support workers.

**Objectives:**

We aim to give an overview of reviews about the effects of peer support work in psychiatric settings and its implementation. Special emphasis on implications for forensic psychiatric settings shall be given.

**Methods:**

Five electronic databases and archives of four relevant journals were searched in December 2019 and updated in April 2022. In addition, references of articles were searched and relevant authors were contacted for unpublished data. Results of reviews were clustered by one author and checked by another.

**Results:**

22 reviews were identified of which 15 reported on effects of peer support work and six on factors influencing its implementation and one review on both. Several effects of peer support work on clinical, psychosocial, organizational and other outcomes (e.g., cost savings) were described. Psychosocial outcomes were the most promising ones whereas no effects were described in most reviews for clinical, organizational and other outcomes. Factors influencing the implementation of peer support work were described during preparation, recruitment, early employment and further development of the peer support worker’s roles. Most factors, beneficial and challenging, were described for the preparation stage of the implementation process.

**Conclusions:**

The authors of the reviews often reported concerns about the low quality of the included studies. Therefore, the present results have to be considered as preliminary. Nevertheless, it is clear that peer support has a positive influence on psychosocial factors and thus complements classic therapeutic approaches. To achieve the best possible effect, the implementation of peer support needs to be carefully planned. Further studies are necessary in order to be able to consider the effect of recovery support in a more differentiated way.

**Disclosure of Interest:**

None Declared

